# Network pharmacology integrated with experimental verification reveals the antipyretic characteristics and mechanism of Zi Xue powder

**DOI:** 10.1080/13880209.2023.2287658

**Published:** 2023-12-09

**Authors:** Hanyu Zhang, Shining Ge, Fengyin Diao, Wen Song, Ying Zhang, Pengwei Zhuang, Yanjun Zhang

**Affiliations:** a College of Chinese Materia Medica, Tianjin University of Traditional Chinese Medicine, Tianjin, China; b Tianjin Hongrentang Pharmaceutical Co., Ltd, Tianjin, China; c State Key Laboratory of Component-based Chinese Medicine, Tianjin University of Traditional Chinese Medicine, Tianjin, China; d Haihe Laboratory of Modern Chinese Medicine, Tianjin, China; e First Teaching Hospital of Tianjin University of Traditional Chinese Medicine, Tianjin, China; f National Clinical Research Center for Chinese Medicine Acupuncture and Moxibustion, Tianjin, China

**Keywords:** Fever, lipopolysaccharide, Chinese medicine, biological network analysis, molecular mechanism

## Abstract

**Context:**

Zi Xue Powder (ZXP) is a traditional formula for the treatment of fever. However, the potential mechanism of action of ZXP remains unknown.

**Objective:**

This study elucidates the antipyretic characteristics of ZXP and the mechanism by which ZXP alleviates fever.

**Materials and methods:**

The key targets and underlying fever-reducing mechanisms of ZXP were predicted using network pharmacology and molecular docking. The targets of ZXP anti-fever active ingredient were obtained by searching TCMSP, STITCH and HERB. Moreover, male Sprague-Dawley rats were randomly divided into four groups: control, lipopolysaccharide (LPS), ZXP (0.54, 1.08, 2.16 g/kg), and positive control (acetaminophen, 0.045 g/kg); the fever model was established by intraperitoneal LPS injection. After the fever model was established at 0.5 h, the rats were administered treatment by gavage, and the anal temperature changes of each group were observed over 10 h after treatment. After 10 h, ELISA and Western blot analysis were used to further investigate the mechanism of ZXP.

**Results:**

Network pharmacology analysis showed that MAPK was a crucial pathway through which ZXP suppresses fever. The results showed that ZXP (2.16 g/kg) decreased PGE2, CRH, TNF-a, IL-6, and IL-1β levels while increasing AVP level compared to the LPS group. Furthermore, the intervention of ZXP inhibited the activation of MAPK pathway in LPS-induced fever rats.

**Conclusions:**

This study provides new insights into the mechanism by which ZXP reduces fever and provides important information and new research ideas for the discovery of antipyretic compounds from traditional Chinese medicine.

## Introduction

Fever is a common clinical symptom in many diseases, especially infection-related diseases. In coronavirus disease 2019 (COVID-19), fever, cough, diarrhea, and malaise are the most common symptoms (Huang et al. 2020). In addition, COVID-19 patients with febrile symptoms have a significantly higher risk of severe illness and mortality than COVID-19 patients without febrile symptoms (Liu et al. 2021). Several studies have reported that in patients with sepsis, fever is associated with poor clinical outcomes and high mortality (Schortgen et al. 2012; Shen et al. 2020; Wu and Lu 2020). In addition, clinical studies have shown that infection is the leading cause of fever in the majority (54–83%) of stroke patients (Stosser et al. 2021). Moreover, stroke patients who develop a fever within 24 h of admission have a significantly increased mortality rate within 1 month after stroke (Prasad and Krishnan 2010). Therefore, understanding fever pathology is important for the diagnosis, treatment, and prognosis of patients with infection-related diseases.

Currently, the clinical treatment of fever includes physical and pharmacological cooling therapies. NSAIDs (e.g., ibuprofen, aspirin, etc.) and acetaminophen are widely used drugs that exert antipyretic and anti-inflammatory effects by inhibiting the synthesis of central prostaglandins (Nguyen et al. 2020). However, some studies have suggested that ibuprofen is not appropriate in younger infants, citing a higher risk of kidney injury, especially in dehydrated children (Tan et al. 2020; Ziesenitz et al. 2022). In this context, it is crucial to develop an effective antipyretic drug with few adverse effects.

Chinese medicine has been used to treat fever for many years. Zi Xue Powder (ZXP) was recorded in the book *Qian Jin Yi Fang* as an important formula for the treatment of febrile seizures in children (Zhang et al. 2022c). ZXP consists of 16 components, including *Glycyrrhizae Radix et* Rhizoma (*Glycyrrhiza uralensis* Fisch. [Leguminosae]), *Aquilariae Lignum Resitum* (*Aquilaria sinensis* [Lour.] Gilg [Thymelaeaceae]), *Aucklandiae Radix* (*Aucklandia lappa* Decne. [Compositae]), *Caryophylli Flos* (*Syzygium aromaticum* [L.] Merrill et. Perry [Myrtaceae]), *Scrophulariae Radix* (*Scrophularia ningpoensis* Hemsl. [Scrophulariaceae]), *Cimicifugae Rhizoma* (*Cimicifuga heracleifolia* Kom. [Ranunculaceae]), *Saiga Tatarica* cornu (*Saiga tatarica* L. [Bovidae]), *Bubali* cornu (*Bubalus bubalis* L. [Bovidae]), *Moschus* (*Moschus moschiferus* L. [Moschidae]), Mirabilitum Crystallina (*Calcitum*), Talc (*Talcum*), Magnetite (*Magnetitum*), Saltpeter (*Niter*), Sodium Sulfate (*Natrii Sulfas*), Gypsum (*Gypsum fibrosum*), and Cinnabar (*Cinnabaris*). At the beginning of the outbreak of the novel coronavirus SARS-CoV-2, ZXP was the recommended medication in the ‘Diagnosis and treatment plan for pneumonia caused by novel coronavirus infection (trial version 3)’ issued by China and in many of the provincial Chinese medicine treatment protocols for COVID-19 (Feng et al. 2020; National Health Commission of the People’s Republic of China, National Administration of Traditional Chinese Medicine 2020). Modern pharmacological research shows that ZXP has antipyretic, anti-inflammatory, and anticonvulsant effects (Wang et al. 1998; Li et al. 2016; Zhang et al. 2022a). Although several studies have focused on the antipyretic effects of ZXP, its mechanism of action has not yet been fully elucidated due to its complex composition and numerous targets of action.

Network pharmacology is an emerging discipline that involves the construction of multilayered networks of disease phenotypes, genes, and drugs. The goal of network pharmacology research is to elucidate scientific questions in a multilayered and systematic manner (Hopkins 2008). The rapid development of public health databases has provided a viable tool for network pharmacology to elucidate the molecular mechanisms of herbal medicine (Zhang et al. 2013). An increasing number traditional Chinese medicine (TCM) studies, are using network pharmacology techniques, which provide a foundation and scientific explanation for the efficacy of TCM (Li et al. 2021; Guo et al. 2022). Therefore, through the joint application of network pharmacology and experimental validation, the antipyretic effect of ZXP can be comprehensively evaluated.

To reveal the mechanism by which ZXP exerts antipyretic effects, this study used a network pharmacology approach to predict the active ingredients, potential targets, and signaling pathways of ZXP for the treatment of fever, and the results were validated using molecular docking and animal experiments.

## Materials and methods

### Screening the active ingredients and targets of ZXP

The targets of the active ZXP plant components (*Glycyrrhizae Radix et Rhizoma*, *Caryophylli Flos*, *Aquilariae Lignum Resitum*, *Aucklandiae Radix*, *Scrophulariae Radix*, and *Cimicifugae Rhizoma*) were obtained by searching the ZXP component names in the traditional Chinese medicine systems pharmacology (TCMSP) database and screening with oral bioavailability (OB) ≥ 30% and drug similarity (DL) ≥ 0.18 (Ru et al. 2014; Yi et al. 2022). For the mineral-based drugs of ZXP (*Calcitum*, *Talcum*, *Magnetitum*, *Niter*, *Natrii Sulfas*, *Gypsum fibrosum,* and *Cinnabaris*), the drug composition information was obtained from the literature (Zhang et al. 1986; Yang et al. 2002; Wu et al. 2010; Song et al. 2020; Zhang and Liu 2021; Pi et al. 2022), and the mineral drug-related genes were obtained using the STITCH database with the selection of *Homo sapiens* and a combined score ≥ 0.4 (Kuhn et al. 2010). Moreover, the composition of the animal components (*Saiga Tatarica* cornu, *Bubali* cornu, and *Moschus*) were obtained using a literature search and the HERB database, their canonical SMIES (CAS) numbers were found using the PubChem database (Yu 2019; Fang et al. 2021). The CAS number was entered into the Swiss Target Prediction database to predict the genes related to the animal-derived components (Gfeller et al. 2014; Kim et al. 2021).

### Predicting targets related to fever

The keywords ‘fever’ and ‘increased body temperature’ were searched in the Online Mendelian Inheritance in Man (OMIM), GeneCards and DrugBank databases as disease targets (Safran et al. 2010; Amberger et al. 2015; Wishart et al. 2018). ZXP and fever-related genes were interactively analyzed using a Venn diagram, and the intersecting genes were the target genes of ZXP for fever treatment.

### Constructing the protein-protein interaction (PPI) network and drug-compound-disease-target network

The intersecting genes were imported into the STRING database, the species was restricted to *Homo sapiens* (von Mering et al. 2003). The genes with interaction scores greater than 0.4 were selected. Subsequently, the PPI network was analyzed using Cytoscape v3.9.1 and calculated topological indices, including median centrality (BC), closeness centrality (CC), and degree. The genes greater than the 2-fold degree value, 1-fold CC value, and 1-fold BC value median were considered core targets. The active components and intersecting genes of ZXP were introduced into Cytoscape v3.9.1 to construct the drug-component-disease network, and the core components for the treatment of fever were screened from the network.

### Gene ontology (GO) and Kyoto encyclopedia of genes and genomes (KEGG) pathway analysis

The top 10 core targets were imported into the DAVID database for GO and KEGG pathway analysis (Dennis et al. 2003). The restricted species was *Homo sapiens*, and the results were screened at *p* < 0.05. Subsequent analysis results were uploaded to the bioinformatics platform for visualization.

### Molecular docking

In this study, the interactions between the core targets and the active compounds were analyzed using molecular docking. The 2D structures of the active components were first downloaded from the PubChem database, and the component structures were defined for energy minimization using Chem 3D software. Then, the protein 3D structure of the core target was downloaded from the Research Collaboratory for Structural Bioinformatics (RCSB) Protein Data Bank (PDB) database (Berman et al. 2000). The water molecules and original ligands were removed by PyMol 2.5 software and transferred to pdbqt format *via* OpenBabel 2.4.1. Next, AutodockTools 1.5.7 was used to identify docking pockets, grid box coordinates and size are set according to the target protein. Subsequently, AutoDock Vina 1.1.2 was employed for molecular docking to evaluate the binding affinity between core potential targets and core compounds. The best-scoring pose as judged by the Vina docking score was chosen and visually analyzed using PyMoL 2.4.0 and Ligplot+ 2.5.5 software.

### Preparation of drugs

ZXP was purchased from Tianjin Hongrentang Pharmaceutical Co., Ltd. (Tianjin, China, H27002). Acetaminophen was purchased from Sino-American Tianjin SmithKline and French Lab., and 0.045 g/kg was equivalent to the clinical equivalent dose. These two drugs were dissolved in saline and sonicated at room temperature for 5 min.

Drug dose selection: In the preliminary animal experimental study, we divided the drug into three dose groups: the low-dose group (0.54 g/kg), medium-dose group (1.08 g/kg) and high-dose group (2.16 g/kg), which represent 1-, 2- and 4-fold dilutions of the clinical dose, respectively, according to the Pharmacopoeia of People’s Republic of China (Commission CP 2020).

### Animal feeding and administration

SPF-grade male Sprague–Dawley (SD) rats, weighing 200–220 g, were purchased from Beijing Vital River Laboratory Animal Technology Co., Ltd. (Beijing, China. License approval number: SCXK 2016-0011). They were housed in a SPF-grade barrier room in the animal center of Tianjin University of Traditional Chinese Medicine at a temperature of 21 ± 2 °C and constant air pressure, alternating between day and night for 12 h. The study protocol was approved by the Animal Ethics Committee of Tianjin University of Traditional Chinese Medicine (ethics committee number TCM-LAEC2020077). This study was conducted in accordance with the guidelines of the Guide for the Care and Use of Laboratory Animals. All efforts were made to alleviate animal suffering and to reduce the number of animals used. The anal temperature of SD rats was measured at 9:00 am each day for 3 days prior to modeling using an electronic thermometer (the electronic thermometer was coated with glycerol and inserted into the rat’s anus at approximately 2 cm). Rats with body temperature fluctuations greater than 1 °C and higher than 38 °C were excluded.

On the day of the experiment, rats were injected intraperitoneally with 0.3 mg/kg lipopolysaccharide (LPS) solution (Sigma-Aldrich, USA, O55:B5, L2880) to establish the fever model, and rats with an anal temperature increase of 0.5 °C within 0.5 h were regarded as successful modeling. In the normal control group, five rats were injected intraperitoneally with saline only. Then, the modelled rats were randomly divided into the LPS group, ZXP low-, medium- and high-dose groups (0.54, 1.08, 2.16 g/kg) and positive control group (acetaminophen, 0.045 g/kg), *n* = 5. The modelled rats were injected with LPS solution for 0.5 h and then intragastrically (i.g) administered the appropriate ZXP and acetaminophen. The anal temperature of each group of rats was measured continuously at 0.5, 1.5, 2.5, 3.5, 4.5, 5.5, 6.5, 8.5, and 10.5 h after modeling, and the temperature rise curves were plotted. After the experiments were completed, rats were sacrificed using isoflurane overdose.

### Enzyme linked immunosorbent assay (ELISA)

ELISA kits were used to detect the levels of PGE2, arginine vasopressin (AVP), and corticotrophin release factor (CRH) in the hypothalamus and the levels of albumin (ALB), TNF-α, IL-6, and IL-1β in the serum of each group of rats. According to the instructions, the optical density (OD) values at 450 nm were detected using an enzyme standardization instrument, and the standard curves of each index were measured and plotted. The level of each sample was calculated by referring to the standard curve. The AVP (JYM0888Ra), CRH (JYM0425Ra), TNF-α (JYM0635Ra), and IL-6 (JYM0646Ra) ELISA kits were purchased from Wuhan Gene Beauty Biotechnology Co., Ltd. The PGE2 (MM-0068R1), ALB (MM-20822R2), and IL-1β (MM-0047R1) ELISA kits were purchased from Jiangsu Enzyme Immune Industrial Co., Ltd.

### Western blot assay

Hypothalamic tissue was lysed using RIPA lysis solution (Solarbio Science & Technology, Beijing, China) and protein loading buffer was prepared after quantification of total protein concentration with a BCA kit (Beyotime Biotech Inc., Shanghai, China). Each group of protein samples was sampled with 20 μg of protein, 4–12% protein prep gel (Jinsirui Biotechnology, Jiangsu, China) was used for constant voltage electrophoresis, and the blots were transferred to PVDF membrane (Millipore, USA) under constant current conditions. The blots were incubated with the blocking solution (Beyotime Biotech Inc., Shanghai, China) for 20 min at room temperature. Then, the blots were incubated with phospho-SAPK/JNK (CST, #4668, 1:1000), SAPK/JNK (CST, #9252, 1:1000), phospho-p38 MAPK (CST, #4511, 1:1000), p38 MAPK (CST, #8690, 1:1000), p44/42 MAPK (CST, #4695, 1:1000), phospho-p44/42 MAPK (CST, #4370, 1:1000), and β-actin (Abcam, ab8226, 1:1000) antibodies overnight at 4 °C. The PVDF membrane was washed the next day using TBST for 5 min/5 times. After incubating the anti-rabbit IgG (CST, #7074, 1:3000) and anti-mouse IgG + IgM (Abcam, ab47827, 1:5000) with PVDF membrane for 1 h at room temperature, the PVDF membrane was washed using TBST for 5 min/5 times. Finally, Immobilon chemiluminescent HRP substrate (Millipore, USA) was added to the PVDF membrane for detection. Grayscale values were measured using ImageJ software.

### Statistical analysis

The experimental data were expressed as the mean ± standard deviation (SD), and SPSS21.0 was used to process the data. One-way ANOVA was used to test for significance between groups, and *p* < 0.05 indicated significant differences. All data were plotted using GraphPad Prism 8.0 software and grouped using Adobe Illustrator CS6 software.

## Results

### Screening of fever-related targets of ZXP

**Figure 1.**
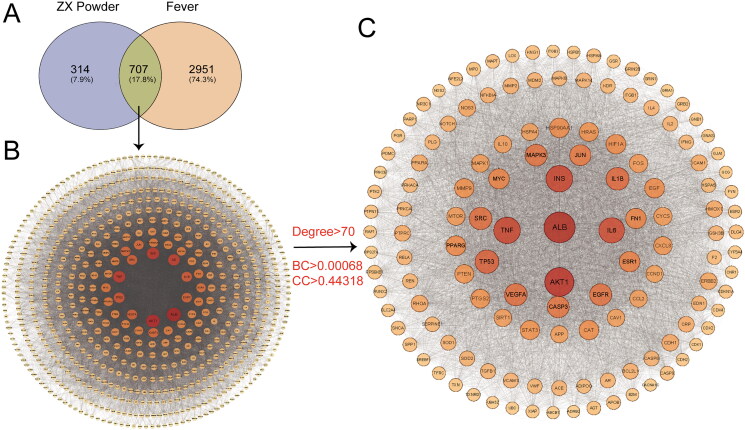
Screening of fever-related targets for ZXP. (A) ZXP and fever-related targets; (B) Protein-protein interaction network of common ZXP and fever-related targets. Node, target protein; Line, interaction between; (C) The core target interaction network. DC: degree; BC: betweenness; CC: closeness. Node, target protein; Line, interaction between.

The 126 components of ZXP with1021 targets were obtained through the TCMSP, STITCH, and Swiss Target Prediction databases. In addition, 3658 fever-related targets were obtained from the OMIM, Gene Cards, and Drug Bank databases. The Venn diagram showed a total of 707 intersecting targets between potential ZXP targets and fever-related targets, which were the targets of ZXP for fever treatment (Figure 1A).

To explore the possible mechanism of ZXP antipyretic activity, we imported the gene names of the 707 fever-related ZXP targets into the STRING database to construct PPI networks. The PPI network of these targets was constructed using Cytoscape v3.9.1 and had 703 nodes and 3438 edges (Figure 1B). The PPI network was further analyzed using the 2-fold degree median (degree > 70), 1-fold BC median (BC > 0.00068), and 1-fold CC median (CC > 0.44318) as the criteria. A total of 132 significant targets were obtained (Figure 1C). The size and color intensity of the node correspond to the degree and probability of being a core target. To obtain the core targets of ZXP for the treatment of fever, we identified the top ten ranked important targets (Figure 1C) by using degree, BC, and CC screening conditions (Table 1). The results suggested that these targets are significantly involved in the antipyretic effect of ZXP.

**Table 1.** Top ten targets in the PPI network.

Name	Degree	Betweenness centrality	Closeness centrality
ALB	332	0.07661953	0.65302326
AKT1	303	0.04122006	0.63300271
INS	271	0.03609976	0.61471103
TNF	270	0.02481293	0.60831889
IL-6	257	0.02128147	0.59897611
TP53	228	0.01910602	0.58548791
IL1B	218	0.01218191	0.57825371
SRC	213	0.01621772	0.58257261
VEGFA	212	0.00908133	0.57259380
EGFR	207	0.01395101	0.57635468

### Drug-compound-disease-target network

**Figure 2.**
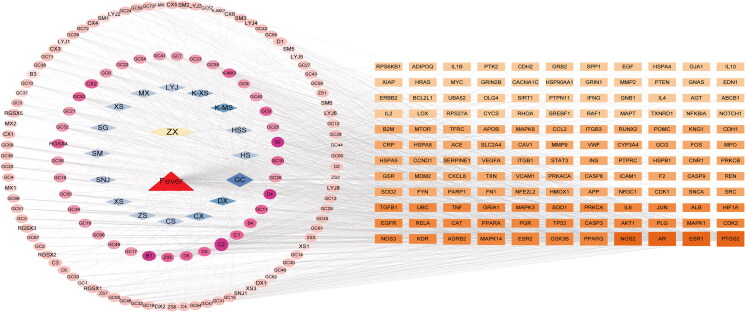
Drug-compound-disease-target network. The yellow hexagon represents ZXP. The red triangle represents fever. The blue diamond represents the formula composition of ZXP. The pink circles represent active compounds contained in ZXP. The orange squares represent the targets.

Given that ZXP contains multiple components, this study used Cytoscape v3.9.1 to construct a drug-compound-disease target network (Figure 2) to further elucidate the main active components of ZXP in fever treatment. The analysis of this network showed that the network contained 275 nodes and 1164 edges. In order to obtain potential fever-treating ZXP compounds, we used the average shortest path length, degree, BC, and CC as screening conditions. The top ten core compounds were screened as quercetin, zinc, kaempferol, licochalcone A, naringenin, 6,7-dimethoxy-2-(2-phenylethyl) chromone, prasterone, formononetin, 7-methoxy-2-methyl isoflavone, and calycosin (Table 2). Therefore, these ZXP compounds were considered effective in the treatment of fever.

**Table 2.** Top ten compounds in the drug-compound-disease-target network.

Lable	Compound	Degree	Average shortest path length	Betweenness centrality	Closeness centrality	Herb
C2	Quercetin	64	2.173145	0.093543	0.460163	*Glycyrrhizae Radix* et Rhizoma, *Aquilariae Lignum Resitum*, *Caryophylli Flos*
B2	Zinc	29	2.505300	0.023761	0.399154	Cinnabaris, Natrii Sulfas
B1	Kaempferol	23	2.519435	0.016478	0.396914	*Glycyrrhizae Radix* et Rhizoma, *Caryophylli Flos*
GC53	Licochalcone A	15	2.590106	0.006353	0.386085	*Glycyrrhizae Radix* et Rhizoma
GC11	Naringenin	14	2.590106	0.008602	0.386085	*Glycyrrhizae Radix* et Rhizoma
CX2	6,7-Dimethoxy-2-(2-phenylethyl) chromone	14	2.604240	0.002731	0.383989	*Aquilariae Lignum Resitum*
SX4	Prasterone	14	2.611307	0.008697	0.382950	artificial moschus
GC9	Formononetin	13	2.611307	0.004347	0.382950	*Glycyrrhizae Radix* et Rhizoma
GC8	7-Methoxy-2-methyl isoflavone	11	2.625442	0.000926	0.380888	*Glycyrrhizae Radix* et Rhizoma
GC10	Calycosin	11	2.625442	0.000926	0.380888	*Glycyrrhizae Radix* et Rhizoma

### Bioinformatics analysis of ZXP-enriched pathways and biological processes

**Figure 3.**
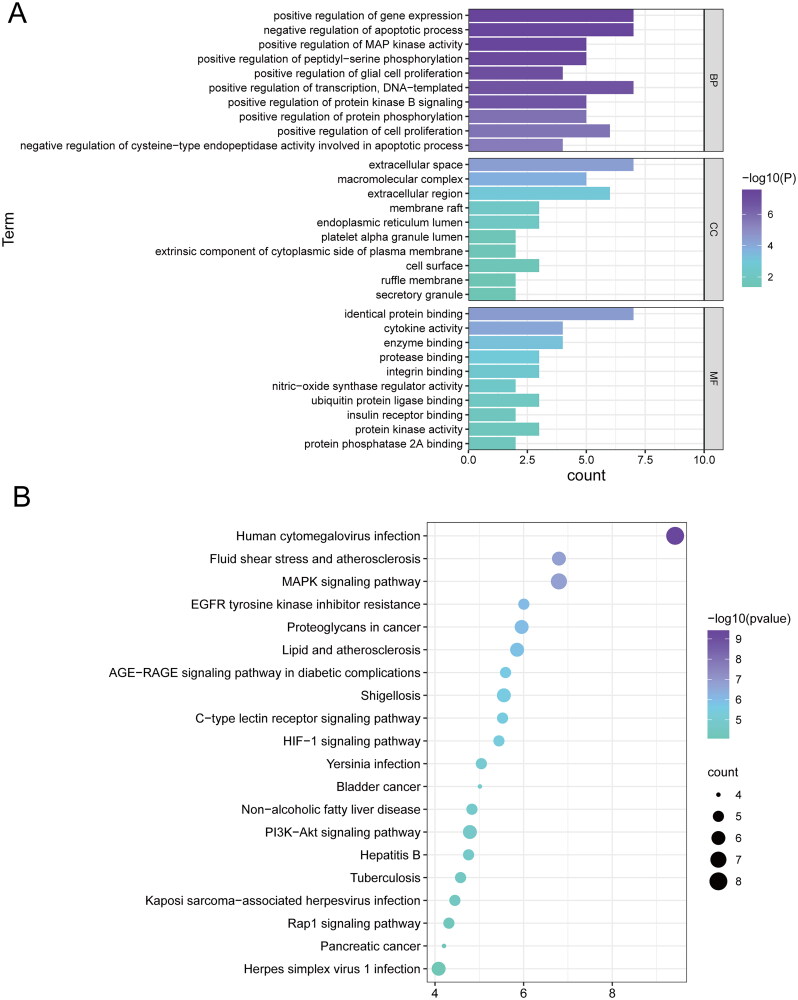
Bioinformatics analysis of ZXP-enriched pathways and biological processes. (A) Top 10 GO terms enriched in core genes; (B) Top 20 KEGG pathways enriched in core genes.

To further explore the multiple antipyretic mechanisms of ZXP, we used the DAVID database to analyze the enriched biological process (BP), cellular component (CC) and molecular function (MF) categories of the 10 core targets. A total of 334 GO terms were enriched (*p* < 0.05), including 171 BP terms, 12 CC terms and 22 MF terms. The top ten GO analysis terms were filtered under each item by p value (Figure 3A). The results showed that in the BP category, ZXP mainly affected gene expression, apoptotic processes, transcription, cell proliferation, and protein phosphorylation. Moreover, ZXP was closely associated with the extracellular gap, extracellular region, macromolecular compounds, membrane rafts, and endoplasmic reticulum lumen in the CC category. In the MF category, the targets were correlated with protein binding, cytokine activity, enzyme binding, protease binding, and integrin binding.

In addition, we performed KEGG pathway enrichment analysis on 10 core targets (*p* < 0.05), and a total of 103 pathways were identified. The top 20 signaling pathways were selected (Figure 3B). The results showed that these targets were mainly enriched in *human cytomegalovirus* infection, *Yersinia pestis* infection, herpes simplex virus type 1 infection, Kaposi sarcoma-associated herpesvirus infection, the MAPK signaling pathway, shigellosis, the C-type lectin receptor signaling pathway, and HIF-1 signaling pathway. The majority of the enriched signaling pathways were associated with bacterial/viral infection and inflammation, suggesting that ZXP may treat fever by interfering with infection and inflammation-related signaling pathways.

### Molecular docking results of the main chemical components of ZXP

**Figure 4.**
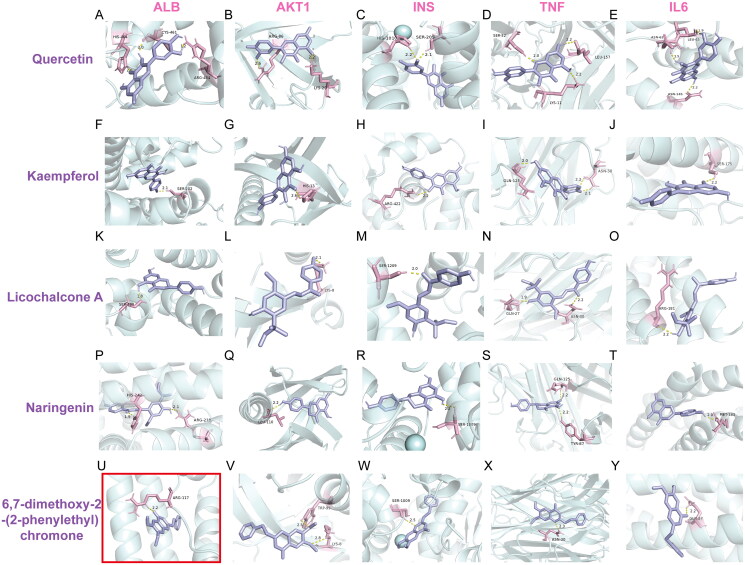
Molecular docking results of the main chemical components of ZXP. (A–E) Quercetin - ALB; Quercetin - AKT1; Quercetin - INS; Quercetin - TNF; Quercetin - IL-6; (F–J) Kaempferol - ALB; Kaempferol - AKT1; Kaempferol - INS; Kaempferol - TNF; Kaempferol - IL-6; (K–O) licochalcone A - ALB; licochalcone A - AKT1; licochalcone A - INS; licochalcone A - TNF; licochalcone A - IL-6; (P–T) Naringenin - ALB; Naringenin - AKT1; Naringenin - INS; Naringenin - TNF; Naringenin - IL-6;(U–Y) 6,7-dimethoxy-2-(2-phenylethyl) chromone - ALB; 6,7-dimethoxy-2-(2-phenylethyl) chromone - AKT1; 6,7-dimethoxy-2-(2-phenylethyl) chromone - INS; 6,7-dimethoxy-2-(2-phenylethyl) chromone - TNF; 6,7-dimethoxy-2-(2-phenylethyl) chromone - IL-6. The potent highest combination of molecular docking was highlighted with a red border.

The network pharmacology results showed that the antipyretic effect of ZXP was associated with anti-infection and anti-inflammation signaling pathways. To further validate the binding ability of the active compounds to the core targets, we performed molecular docking experiments. The core components of ZXP exert their antipyretic effects by affecting the core targets. Therefore, the top five core targets (associated with inflammation and infection) were selected for molecular docking with the top five potential compounds (excluding the metal components) in the drug-component-disease target network. Using AutoDockTools-1.5.6 and AutoDock Vina-1.1.2, the interactions between the five core targets and the five active chemicals were examined. The binding energies of the core targets and the compounds are shown in Table 3. The outcomes of the molecular docking were shown using PyMOL (Figure 4).

**Table 3.** The binding energy of the compounds and core targets (kcal/mol).

Target	Target (PDB ID)	Target structure	Compound	Affinity (kcal/mol)
ALB	1N5U	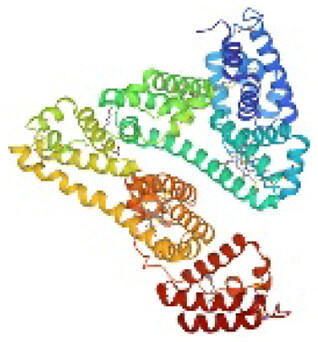	Quercetin	−8.1
			Kaempferol	−7.0
			Licochalcone A	−8.0
			Naringenin	−8.5
			6,7-Dimethoxy-2-(2-phenylethyl) chromone	−9.6
AKT1	1H10	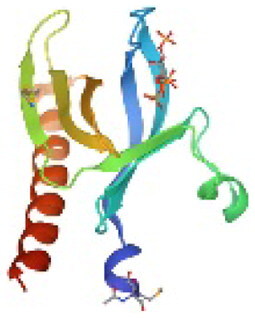	Quercetin	−6.1
			Kaempferol	−6.0
			Licochalcone A	−6.3
			Naringenin	−6.1
			6,7-Dimethoxy-2-(2-phenylethyl) chromone	−6.2
INS	1HTV	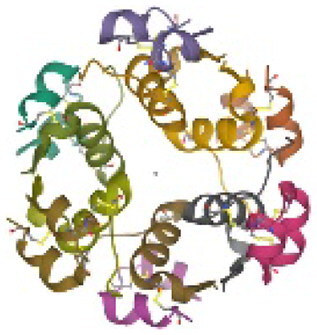	Quercetin	−7.9
			Kaempferol	−5.8
			Licochalcone A	−6.6
			Naringenin	−7.8
			6,7-Dimethoxy-2-(2-phenylethyl) chromone	−8.6
TNF	1TNF	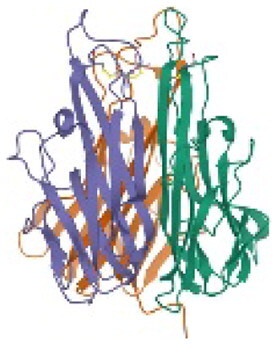	Quercetin	−6.3
			Kaempferol	−6.4
			Licochalcone A	−5.5
			Naringenin	−6.8
			6,7-Dimethoxy-2-(2-phenylethyl) chromone	−5.4
IL6	7NXZ	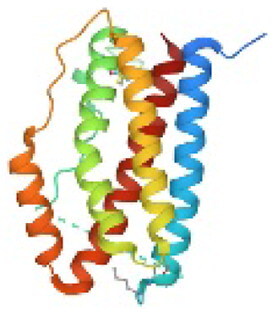	Quercetin	−6.4
			Kaempferol	−6.8
			Licochalcone A	−5.2
			Naringenin	−5.3
			6,7-Dimethoxy-2-(2-phenylethyl) chromone	−5.4

The results showed that quercetin might interact with IL-6, ALB, Serine/threonine kinase 1 (AKT1), insulin (INS), and TNF (Figure 4A–E). According to Figure 4A, quercetin bound to HIS-464, CYS-461, and ARG-484 in ALB by a single hydrogen bond. Figure 4B shows that ARG-86 and LYS-20 in AKT1 and quercetin were linked through a single hydrogen bond. Quercetin and HIS-1010 and SER-209 in INS were bound by a single hydrogen bond, as shown in Figure 4C. SER-52, LEU-157, and LYS-11 in TNF were shown to have formed a single hydrogen bond with quercetin in Figure 4D. According to Figure 4E, quercetin joined IL-6, ASN-62, ASN-143, and LEU-63 by a single hydrogen bond.

The results demonstrated that kaempferol might interact with IL-6, INS, TNF, ALB, and AKT1 (Figure 4F–J). Kaempferol and SER-202 in ALB were bound by one hydrogen bond, as shown in Figure 4F. According to Figure 4G, kaempferol and HIS-13 in AKT1 can interact through a single hydrogen bond. In addition, kaempferol and INS created one hydrogen bond (Figure 4H). In TNF, kaempferol established two hydrogen bonds with ASN-30 and one with GLN-125, as shown in Figure 41. Additionally, one hydrogen bond was created between kaempferol and IL-6, as shown in Figure 4J.

The results showed that licochalcone A could interact with ALB, AKT1, INS, TNF, and IL-6 (Figure 4K–O). Figure 4K shows that licochalcone A can interact with HIS-242 and ARG-218 in ALB through one hydrogen bond. As shown in Figure 4L, licochalcone A formed one hydrogen bond with LSY-8 in AKT1. Figure 4M shows that licochalcone A can interact with SER-1209 in INS through one hydrogen bond. Furthermore, licochalcone A formed one hydrogen bond with GLN-27 and ASN-30 in TNF (Figure 4N). As shown in Figure 4O, one hydrogen bond was formed between licochalcone A and IL-6.

The findings demonstrated that naringenin might interact with IL-6, ALB, AKT1, INS, and TNF (Figure 4P–T). Figure 4P shows that naringenin formed one hydrogen bond with SER-480 in ALB. Figure 4Q shows that naringenin formed one hydrogen bond with LEU-110 in AKT1. Figure 4R shows that naringenin formed one hydrogen bond with SER-1009 in INS. Figure 4S shows that naringenin formed one hydrogen bond with GLN-125 and TYR-87 in TNF. Figure 4T shows that naringenin formed one hydrogen bond with MET-183 in IL-6.

The results showed that 6,7-dimethoxy-2-(2-phenylethyl) chromone could interact with ALB, AKT1, INS, TNF, and IL-6 (Figure 4U–Y). As shown in Figure 4U, 6,7-dimethoxy-2-(2-phenylethyl) chromone formed one hydrogen bond with ARG-117 in ALB. Figure 4V shows that 6,7-dimethoxy-2-(2-phenylethyl) chromone can interact with TRP-99 and LYS-8 in AKT1 through one hydrogen bond. In addition, one hydrogen bond was formed between 6,7-dimethoxy-2-(2-phenylethyl) chromone and INS (Figure 4W). Figure 4X shows that 6,7-dimethoxy-2-(2-phenylethyl) chromone formed one hydrogen bond with ASN-30 in TNF, and two hydrogen bonds with ASN-30. As shown in Figure 4Y, one hydrogen bond was formed between 6,7-dimethoxy-2-(2-phenylethyl) chromone and IL-6.

To assess the complementarity between the compound and protein, the binding energy was calculated (Li et al. 2022). Lower binding energy corresponds to high stability. Generally, a binding energy less than −5 kcal/mol indicates favorable binding activity between the ligand and the receptor. The molecular docking results showed that these binding energies were all less than −5 kcal/mol, indicating that the compounds had high affinity for the proteins (Table 3). The binding energy of ALB docked with 6,7-dimethoxy-2-(2-phenylethyl) chromone was the highest (-9.6 kcal/mol), and the binding energy of IL-6 docked with licochalcone A was the lowest (-5.2 kcal/mol). These five compounds (quercetin, kaempferol, licochalcone A, naringenin, and 6,7-dimethoxy-2-(2-phenylethyl) chromone) bound well to the five core targets (ALB, AKT1, INS, TNF, and IL-6). Therefore, we predicted that these compounds may play a key role in fever treatment.

### Antipyretic effects of ZXP verified by in vivo experiments

**Figure 5.**
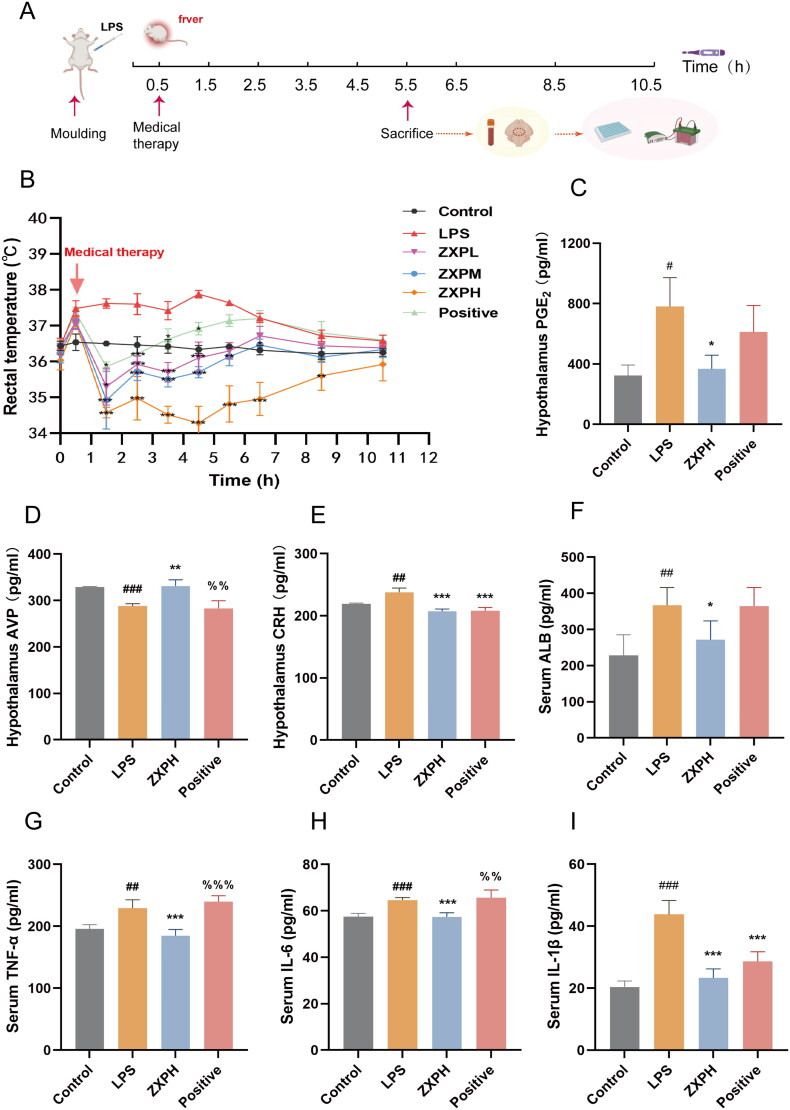
The effects of ZXP on rats with LPS-induced fevers. (A) The timeline of experimental modeling and drug administration; (B) The effects of different doses of ZXP on the body temperature of rats with LPS-induced fever; ZXP regulated the levels of the hypothalamic thermoregulatory factors PGE2 (C), AVP (D), and CRH (E) in LPS-induced fever rats; ZXP regulated the levels of serum ALB (F), TNF-a (G), IL-6 (H), and IL-1β (I) in LPS-induced fever rats. ^#^*p* < 0.05, ^##^*p* < 0.01, ^###^*p* < 0.001 in comparison with the control group. *p < 0.05, **p < 0.01, ***p < 0.001 in comparison with the LPS group.

After the network pharmacological analysis, a rat model of LPS-induced fever was established to verify the potential molecular mechanism by which ZXP exerts its antipyretic effect (Figure 5A). As shown in Figure 5B, the body temperature of the LPS-induced model group showed a significant upward trend at 0.5 h after induction, reaching a peak at 4.5 h, and then the increase in body temperature gradually slowed. Acetaminophen is a commonly used antipyretic agent and was used as a positive control drug to reduce the body temperature of LPS-induced fever rats at 0.5–5.5 h; however, the body temperatures of rats in the acetaminophen and LPS groups were similar at 5.5–10.5 h. In contrast to the acetaminophen group and LPS group, the high-dose ZXP group showed significantly lower body temperatures from 0.5 to 8.5 h. The results showed that ZXP had significant inhibitory effects on LPS-induced fever in rats in a dose-dependent manner, and that antipyretic effect of the high-dose ZXP group was the most effective. Among the many modulators of body temperature, PGE2, AVP, and CRH are important thermoregulatory factors (Telegdy and Adamik 2008; Huang et al. 2020). We used the high-dose ZXP treatment group to evaluate the levels of PGE2, AVP and CRH in the hypothalamic tissue of rats 5.5 h after modeling and the antipyretic pharmacological effect of ZXP (Figure 5C–E). Compared with the control group, the LPS group exhibited significantly increased serum PGE2 and CRH levels, while serum AVP levels were significantly decreased. Compared the LPS-treated rats, the high-dose ZXP treated rats showed a significant decrease in PGE2 and CRH levels, and high-dose ZXP treatment caused an increase in AVP levels. The hypothalamic CRH level was significantly lower in the positive control group than in the LPS group, while the hypothalamic PGE2 and AVP levels in the positive control group were similar to those in the LPS group.

**Figure 6.**
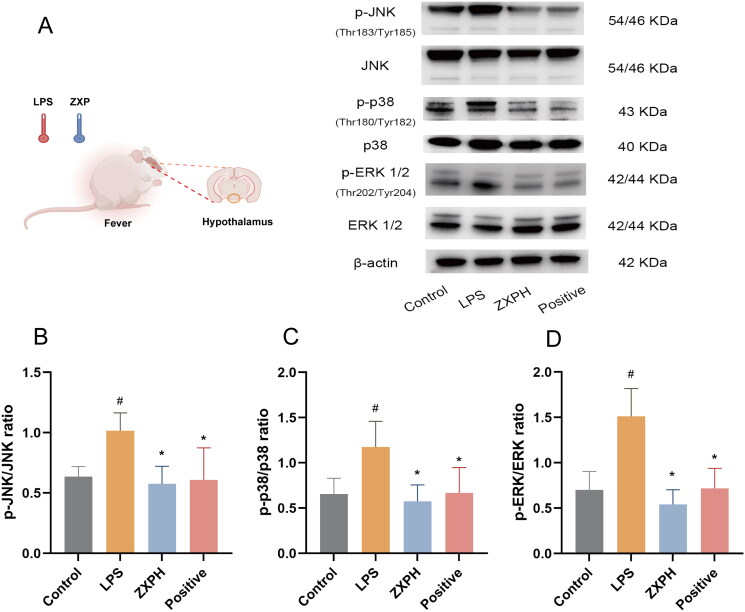
ZXP inhibited LPS-induced MAPK signaling in rats. (A) Western blotting assays were performed to detect the expression levels of p-JNK, JNK, p-p38, p38, p-ERK 1/2, and ERK 1/2. The p-JNK/JNK (B), p-p38/p38 (C) and p-ERK 1/2/ERK 1/2 (D) were calculated by grayscale analysis. ^#^*p* < 0.05 in comparison with the control group. *p < 0.05 in comparison with the LPS group.

Several core genes in network pharmacology analysis were screened out for further validation, including inflammation-related ALB, TNF-a, IL-6, and IL-1β. First, ELISA was used to confirm the levels of the core genes. Our results showed that serum ALB, TNF-a, IL-6, and IL-1β levels were significantly higher in the LPS group than in the control group. Indeed, it was found that the levels of serum ALB, TNF-a, IL-6, and IL-1β decreased in fever-induced rats treated with high-dose ZXP (Figure 5F–1). However, compared to the LPS group, the positive control group only exhibited significantly reduced serum IL-1β levels. Furthermore, an immunoblotting assay was performed to explore the MAPK signaling pathway as a potential pathway for ZXP treatment of fever. As a result, high-dose ZXP was found to inhibit the MAPK pathway by decreasing the protein expression levels of p-JNK, p-p38, and p-ERK 1/2 (Figure 6A–D). Overall, these results indicated that inflammation is the crucial mechanism of action by which ZXP suppresses fever progression.

## Discussion

The antipyretic effect of ZXP has been known for thousands of years. It has been previously recorded in the Pharmacopoeia of the People’s Republic of China that ZXP could treat fever (Commission CP 2020). However, due to the complex chemical components of ZXP, it remains difficult to elucidate its potential active compounds and precise pharmacological mechanisms in treating fever.

In this study, we conducted a systemic study using a combination of network pharmacology and experimental verification to elucidate the bioactive components and therapeutic mechanisms of ZXP in fever. Fever is a signal of infectious and inflammatory diseases. LPS enters the circulation and triggers TLR-4, which induces the transcription of COX-2 into PGE2 and induces fever (Salvi et al. 2016). Moreover, LPS also activates a pyrogenic factor located on circumventricular organs (CVOs), which leads to the release of PGE2 and caused fever (Ma et al. 2021). Here, we found that ALB, AKT1, INS, TNF-α, and IL-6 were the core target proteins of ZXP for the treatment of fever. ALB, AKT1, INS, TNF-α, and IL-6 can influence the fever process by affecting the generation and central transport of PGE2. The synthesized PGE2 in the periphery binds to ALB to form a complex, and the complex is transported through the circulation to the temperature center to initiate early fever (Romanovsky et al. 1999; Ivanov et al. 2005). AKT1 is the critical enzyme of the PI3K/AKT signaling pathway, which is involved in inflammation and activates IκB kinase (IKK), which in turn leads to the activation of the NF-κB pathway (Jackson-Bernitsas et al. 2007). The NF-κB pathway upregulates the expression of inflammatory mediators such as TNF-α, IL-1, iNOS, and COX-2 (Jackson-Bernitsas et al. 2007; Chen et al. 2009; Li et al. 2019). COX-2 catalyzes the synthesis of PGE2 and is involved in the febrile response (Yang et al. 2020; Zhang et al. 2022b). INS is a critical hormone in maintaining the physiological response to glucose, and it can reduce inflammation by regulating the activation of the NOD-like receptor protein 3 (NLRP3) inflammasome (Chang et al. 2020). Moreover, studies have shown that INS may act synergistically with LPS to induce the production of cytokines and PGE2 (Klauder et al. 2020). TNF-α and IL-6 are pyrogenic cytokines that are secreted in the blood after LPS recognition and directly or indirectly affect the production of PGE2 (Roth and Blatteis 2014). IL-6 activates the STAT3 pathway by binding to its receptor on brain endothelial cells to induce COX-2 expression to produce PGE2 (Eskilsson et al. 2014). TNF-α activates mPGES-1 and produces PGE2 under a glutathione-mediated signaling pathway (Wrotek et al. 2015; Ma et al. 2021). In addition, pyrogen or pyrogenic cytokines activate the complement system to release complement component C5a (C5a), which further triggers the activation of COX-1 by Kupffer cells (Kc) to catalyze PGE2 production (Perlik et al. 2005). In addition to PGE2, the hypothalamus regulates body temperature by secreting the thermoregulatory factors CRH and AVP. Animal experiments confirmed that intracerebroventricular injection of CRH in rats induced significant fever and brown adipogenic heat (De Souza et al. 2002). In contrast to CRH and PGE2, AVP is a central endogenous neurotransmitter that negatively regulates body temperature during fever (Huang et al. 2020). The present study also confirmed that ZXP could regulate the levels of PGE2, CRH, and AVP in the hypothalamus, which was consistent with the findings in the literature. The predicted performances from network pharmacology were further confirmed by *in vivo* experiments, suggesting that ZXP mainly exerted its antipyretic effects by inhibiting the production of multiple pyrogenic cytokines (TNF-a, IL-6, and IL-1β) and the gene expression levels of fever mediators (PGE2, CRH, and AVP).

Most of the ZXP compounds in the drug-compound-disease-target network affected multiple targets and many overlapping targets were identified from different compounds, indicating that ZXP plays an antipyretic role through the synergistic effects of its compounds. The predicted active ingredients (quercetin, kaempferol, naringenin, 6,7-dimethoxy-2-(2-phenylethyl) chromone, and licochalcone A) have inhibitory effects on bacterial/viral infection and inflammation signaling pathways (Wang et al. 2018; Fanunza et al. 2020; Arabyan et al. 2021; Santhi et al. 2021; Li et al. 2022). These active components can suppress pyrogenic cytokine production, fever mediator gene expression, and inflammation-related signaling pathways in models of inflammation (Morikawa et al. 2003; Chu et al. 2012; Park et al. 2012; Fu et al. 2013; Khan et al. 2020). These studies provide supportive evidence that quercetin, kaempferol, naringenin, 6,7-dimethoxy-2-(2-phenylethyl) chromone, and licochalcone A have favorable inhibitory effects on pyrogenic cytokines and fever mediators. These compounds may be important for the therapeutic effect of ZXP on fever.

To explore the mechanism by which ZXP treats fever, we conducted GO and KEGG enrichment analyses. The GO results showed that the target genes were mainly enriched in BPs such as gene expression, apoptotic process, transcription, cell proliferation, and protein phosphorylation. KEGG enrichment analysis results showed that ZXP mainly interfered with the occurrence and development of fever through bacterial/viral infection (*human cytomegaloviru* infection, *Yersinia pestis* infection, etc.) and inflammation-related (MAPK, HIF-1C-type lectin receptor, PI3K-AKT, etc.) signaling pathways. The MAPK signaling pathway was highly enriched, and we speculate that this pathway is likely to be the main signaling pathway ZXP regulates to alleviate fever. The MAPK pathway plays an important role in inflammatory responses against pathogenic invasion and is the common pathway for many infectious diseases with febrile features, such as the early acute febrile stage of dengue infection and *Streptococcus pneumoniae* infection (McGuire et al. 2013; Harikrishnan et al. 2018; Bajrai et al. 2021). Moreover, the MAPK signaling pathway is extensively involved in viral replication, fever mediator production, inflammatory response, and cellular stress during infection (Ridder et al. 2011; Sreekanth et al. 2018; Cheng et al. 2020; Liu et al. 2020). Phosphorylation of the extracellular signal-regulated kinases ERK1/2 and p38 promotes the production of TNF-α, IL-6, and PGE2 to increase body temperature (Kirkwood et al. 2003; Blomqvist and Engblom 2018; Wang et al. 2022). Furthermore, we validated the predicted results of the network pharmacology by conducting an *in vivo* experiment, which showed that ZXP interfered with LPS-induced fever *via* inhibition of the MAPK signaling pathway.

Subsequently, we analyzed five core bioactive components (quercetin, kaempferol, licochalcone A, naringenin, and 6,7-dimethoxy-2-(2-phenylethyl) chromone) and five core targets (ALB, AKT1, INS, TNF, and IL-6) of ZXP from the network pharmacological results. Molecular docking of the core components and targets was performed to verify their binding compatibility. The docking results showed that these five compounds could bind to the protein very well, and 6,7-dimethoxy-2-(2-phenylethyl) chromone and AKT1 had the lowest binding energy, indicating that the binding of this active component and target protein combination was the most stable.

This study has some limitations. First, some compounds and target genes may not have been included in public databases. Second, although the results of this study showed that ZXP could alleviate fever in rats, the effects of different combinations and doses of the active components of ZXP on febrile animals need to be verified by further pharmacological experiments.

## Conclusions

The present study first demonstrated that ZXP has an antipyretic effect with long-lasting characteristics, and the antipyretic effect may mainly be attributed to inhibiting the MAPK signaling pathway in hypothalamic tissue and then suppressing the inflammatory response. The present research offers a theoretical foundation for the comprehensive investigation of the therapeutic mechanism of ZXP in the treatment of fever. Additionally, it has provided significant progress in elucidating the pharmacological basis and mechanism of TCM in ameliorating infection and inflammatory-induced fever diseases.

## Data Availability

The data that support the findings of this study are available from the corresponding author upon reasonable request.
